# Letters to the Editor

**Published:** 1912-10

**Authors:** 


### Letters to the Editor.

### FROM DEAR DOCTOR WYETH.

LAKE PLACID CLUB, ESSEX CO., N. Y., Sept. 8, 1912.

MY DEAR DOCTOR DANIEL: I congratulate you on the increased circulation of the JOURNAL, and am pleased with whatever adds to your happiness and prosperity. A man's life may be compared to the ball of snow which as a boy he started from the hill-top. It grew larger with each turn, carried more weight with it, went faster the further it rolled and nothing could stop it until it hit the bottom!

The older I get the busier. You must come up and see the new Polyclinic Hospital. The Polyclinic has always found a true and loyal friend in the JOURNAL, and I one in its genial editor.

Ever your friend,

JOHN A. WYETH.

---

PEORIA HEIGHTS, ILL., August 31, 1912.

*Dr. F. E. Daniel, Editor "Red Back," Austin, Texas.*

DEAR DOCTOR: I found your August number quite interesting. It seems to me that you and Dr. Bogart deserve special credit as pioneering the way in this matter of sterilizing the unfit. I have mentioned you both several times in articles I have contributed to journals. It certainly is a good movement, but, like nearly all such, it is a "labor of love."

I like your JOURNAL for its originality and fearless stand on all important matters appertaining to the profession.

Thanking you for courtesies, I am,

Very truly yours,

W. T. MARRS, M. D.

---

"SWAT THE FLY."

JOHNSON CITY, August 23, 1912.

*Dr. F. E. Daniel, Editor Texas Medical Journal, Austin, Texas.*

DEAR DOCTOR: Now is the time to begin a campaign against that most dangerous of modern pests—the housefly. For the next seven months the number of flies will be gradually diminishing, and this will make it easier to kill off those living during the winter season, and it is especially possible during these months to destroy the breeding places of this pest.

Literature should be spread broadcast over our State telling of the danger of the presence of flies in our midst, and also telling the means of destroying the breeding places during the winter months. By this means we shall be able to destroy many times the number we would during the summer months by simply killing the full-grown insect.

As in my letter to you advocating the plan of physicians publicly conducting a campaign of moral hygiene, the same reasons cover this plan of getting rid of the flies. In this campaign, however, we can better enlist the aid of the general public, as they know some of the dangers of the prevalence of flies and also some of the methods of destroying their winter quarters.

Free literature on this subject, aided by supervision by the medical profession as far as is in their power and is compatible with their willingness to aid in the fight, will result in a great diminution in the number of flies which will infest our State next summer. A set of rules for the destruction of breeding places with an urgent appeal to apply those rules periodically during the winter months, put within the reach of all, is part of a plan which can not help but be of great benefit in this fight.

I have no desire either to dictate to, or embarrass, our State Board of Health by these suggestions; on the contrary, I, together with the rest of the profession, have the greatest confidence in their ability to combat this pest. These suggestions are simply from one who is much interested in the prevention of disease.

Yours fraternally,

GEO. HARWOOD, M. D.

---

TEXAS STATE BOARD OF HEALTH,
DEPARTMENT OF VITAL STATISTICS.

AUSTIN, TEXAS, September 12, 1912.

*Editor "Red Back":*

I beg to thank you, though tardily, for the copies of your excellent JOURNAL. These are the first copies that have been received in this department, and I beg to state that I have certainly been missing a great treat. Feel sure I shall find great aid in its pages toward the making up of our *Bulletin.*

It may interest you to learn of the work I am doing this month. Having prepared a list of all boys 12 years of age (which numbers about 30,000) throughout the State, I am addressing a letter to the parents of same, following said letter with a copy of our August *Bulletin,* whose feature story is a copy of the "Boys Venereal Peril" pamphlet, issued by the American Medical Association, copy of which you have doubtless seen. I think the results of this labor will prove far-reaching and do untold good.

Sincerely yours,

R. P. BABCOCK,
Registrar of Vital Statistics.

